# Assessment of Food Consumption Frequency Among Schoolchildren: Cross-Sectional Study from an Urbanized Region of Central Kazakhstan

**DOI:** 10.3390/nu18172774

**Published:** 2026-08-25

**Authors:** Svetlana Rogova, Zhanerke Bolatova, Karina Nukeshtayeva, Olzhas Zhamantayev, Gaukhar Kayupova, Aza Galayeva, Nurzhamal Shintayeva, Olga Plotnikova, Denis Turchaninov

**Affiliations:** 1 School of Public Health, Karaganda Medical University, Karaganda 100008, Kazakhstan; s.rogova@qmu.kz (S.R.); nukeshtaeva@qmu.kz (K.N.); kayupovag@qmu.kz (G.K.); shintaeva@qmu.kz (N.S.); 2 School of Residency and Professional Development, Karaganda Medical University, Karaganda 100008, Kazakhstan; 3 Department of Occupational Hygiene and Occupational Pathology, Omsk State Medical University, Omsk 644099, Russia; olga.plotnikova7@mail.ru; 4 Department of Hygiene and Human Nutrition, Omsk State Medical University, Omsk 644099, Russia; omskgsen@yandex.ru

**Keywords:** schoolchildren, dietary patterns, food consumption frequency, eating behavior, adolescents

## Abstract

**Background/Objectives**: Nutrition during school age is a key public health issue, as eating habits formed in this period may affect growth, cognitive development, and future health. This study aimed to assess food consumption frequency and age, gender, and socio-demographic differences in dietary patterns among schoolchildren aged 7–17 years in Karaganda, Kazakhstan. **Methods**: A cross-sectional study was conducted among schoolchildren aged 7–17 years attending state secondary schools in Karaganda, Kazakhstan, from September 2025 to February 2026. Food consumption frequency was assessed using a bilingual Russian–Kazakh questionnaire developed by the authors based on food frequency questionnaire approaches and adapted to the Kazakhstani context; the questionnaire included 41 food items, including national meat products and traditional fermented milk drinks, and Principal Component Analysis was applied to identify dietary patterns. **Results**: A total of 864 schoolchildren aged 7–17 years were included in the study, with comparable distribution by sex and age groups. Frequent consumption of fast food, coffee, processed and canned meat products increased markedly with age, while consumption of milk, fermented dairy products, juices, fresh fish and berries was lower, especially among older adolescents. Principal Component Analysis identified three dietary patterns—“processed and fast-food”, “traditional meat-based”, and “mixed dairy-based pattern”—with the processed and fast-food pattern more strongly associated with older age, non-Kazakh ethnicity, and school location in the South-East district. **Conclusions**: The findings indicate age-related shifts toward less balanced dietary choices among Karaganda schoolchildren, particularly among adolescents, supporting the need for targeted school- and family-based interventions to reduce fast food, sugary drink, coffee, and processed meat consumption while promoting healthier food choices.

## 1. Introduction

The problem of nutrition is considered one of the priorities of public health. It becomes especially important at school age, when stable eating habits are formed that can influence the state of health in subsequent age periods. During this period, intensive processes of growth and development of the body occur, accompanied by an increased need for macro- and micronutrients [1,2]. Research shows a link between the nutrition of schoolchildren, cognitive functions, concentration, physical development and academic performance [3].

Dietary disorders and an unbalanced diet are associated with an increased risk of developing obesity, type 2 diabetes, iron deficiency conditions and diseases of the gastrointestinal tract in subsequent age periods [4,5,6].

Recent studies indicate a change in the diet and structure of children and adolescents. According to European studies, only a small proportion of schoolchildren adhere to the recommended regularity of meals. A relationship has been established between the diet, the frequency of snacking and the quality of the diet [7]. More frequent consumption of foods high in sugar and energy, including sugary snacks, as well as irregular meals are associated with a deterioration in the quality of the diet among schoolchildren [8,9].

According to NHANES, frequent meals outside the home are associated with a less balanced diet in adolescents [10]. Studies also point to the association of frequent snacking with increased calorie intake and increased consumption of foods high in sugar and fat [11]. An important feature of the modern adolescent diet is the high proportion of ultraprocessed foods, providing up to 60–65% of daily energy intake [5,12].

Similar changes in the diet and structure of schoolchildren have been described in the countries of East Asia and the CIS, where skipping breakfast, consumption of fast food, sugary drinks, and highly processed foods are noted [8,9].

Studies conducted in Kazakhstan indicate the influence of social and family factors on the eating behavior of schoolchildren. It has been shown that the diet of schoolchildren may vary depending on the social status of the family and living conditions, while the consumption of sweets and sugar-containing beverages is widespread among children and adolescents [5,12].

The assessment of the frequency of consumption of the main food groups is of particular importance, since it allows us to characterize stable nutritional features, rather than single deviations in the diet. Food consumption frequency questionnaires are widely used in studies of children and adolescents to assess their habitual diet, especially when analyzing individual food groups [13,14]. This approach is particularly informative when studying foods whose regular consumption is associated with an increased risk of overweight and metabolic disorders, including sugary drinks and fast food [15,16,17].

However, data on the actual eating behavior of schoolchildren in Kazakhstan, including the frequency of consumption of major food groups, remains limited, especially for urbanized regions and taking into account age, gender, and socio-demographic differences. Central Kazakhstan, in particular the city of Karaganda, is of interest as an urbanized region with pronounced urban heterogeneity, where differences in infrastructure, socio-economic profile of the population and food availability can form different conditions of food choice for schoolchildren [18,19]. Accordingly, the present study aimed to address the following research questions:

1. What is the frequency of consumption of major food groups among schoolchildren aged 7–17 years living in an urbanized region of Central Kazakhstan?
2. Does the frequency of consumption of individual food items and food groups differ according to schoolchildren’s age and sex?
3. Which dietary patterns can be identified based on the frequency of food consumption, and how are these patterns associated with schoolchildren’s sociodemographic characteristics, including school district, ethnocultural background, family socioeconomic characteristics, and school shift?

The purpose of this study was to assess the frequency of consumption of the main food groups among schoolchildren aged 7–17 years living in the urbanized region of Central Kazakhstan, the city of Karaganda, as well as to analyze age, gender, and socio-demographic differences in the structure of nutrition.

## 2. Materials and Methods

### 2.1. Study Design, Sampling Strategy, and Inclusion/Exclusion Criteria

The participants of the cross-sectional study were schoolchildren aged 7–17 years old, studying in state secondary schools in Karaganda, Kazakhstan. Data collection was conducted from September 2025 to February 2026.

Based on official urban planning documents [20], two contrasting districts of Karaganda (Maikuduk and South-East) were selected. In coordination with the city Department of Education, six public schools were selected in each district (12 in total).

During the days of the research group’s visit to schools, the sample consistently included all students present in the classroom who met the selection criteria and provided informed consent. Data collection was conducted only on school days during the school year. For children under the age of 14, data was collected by interviewing parents or legal representatives. Teenagers aged 15–17 answered independently.

Inclusion criteria: study at a selected public school, permanent residence in the Maikuduk or Southeastern area, belonging to one of three age categories: 7–10 years old, 11–14 years old or 15–17 years old.

Exclusion criteria: chronic diseases requiring therapeutic nutrition (according to parents); acute fever at the time of the survey or the day before; diagnosed developmental or behavioral disorders that prevent participation; incomplete questionnaires or fatal data entry errors; refusal to participate at any stage.

### 2.2. Sample Size Calculation and Pilot Phase

The minimum sample size was calculated using the Cochran formula for fractional indicators with a 95% confidence interval, maximum variability of the feature (p = 0.5) and a marginal error of 5% (Z = 1.96), which amounted to n_0_ = 384.

N_0_ = (Z^2^ × p × (1 − p))/e^2^

After adjusting for the final population of Karaganda schoolchildren (50,000), the minimum sample size was 381 people.

Taking into account the cluster structure of the sample at the school level, the design effect (DEFF) = 2.0 was applied, as a result of which the required volume increased to 762 participants [21,22]. Taking into account the possible non-response, the planned sample size was increased. The final analysis included 864 schoolchildren.

Before the main stage of the study, a pilot test of the food consumption frequency questionnaire was conducted on 35 participants not included in the main sample. The clarity of the questions, the completeness of the list of products, the convenience of frequency categories, and the interview procedure in Russian and Kazakh were evaluated. Based on the results of the pilot, the individual formulations and structure of the questionnaire were clarified. The pilot phase data was not included in the main analysis.

### 2.3. Ethical Considerations

Before the start of the interview, each participant signed a voluntary informed consent form and received an explanation of the objectives and procedures of the study. The research protocol was approved by the Ethics Committee of Karaganda Medical University (Protocol No. 16 dated 16 September 2025). The work was carried out in accordance with the Helsinki Declaration. Confidentiality is ensured by assigning each participant a unique code. Access to the database is restricted to members of the research team.

### 2.4. Assessment of the Frequency of Food Consumption and Data Processing

The frequency of food consumption was assessed using a questionnaire developed by the authors, based on FFQ approaches [23] and adapted to take into account the nutrition structure of schoolchildren in Kazakhstan. The questionnaire included 40 food items, including national meat products and traditional fermented milk drinks.

Sugar consumption referred to the use of table sugar primarily for sweetening tea and coffee, while confectionery products and sugar-sweetened beverages were assessed separately.

The respondents indicated the usual frequency of consumption of each product using the categories “daily”, “several times a week”, “several times a month”, “rarely or never”. The questionnaire was used in Russian and Kazakh versions.

At the analysis stage, the items were grouped into 12 enlarged product groups based on the similarity of the food profile: milk and dairy products; fats and fatty products; meat and meat products; fish and fish products; eggs; grain and processed products; confectionery; legumes and nuts; vegetables, mushrooms and processed products; fruits, berries and their processed products; beverages; fast food. The association was used only for analytical interpretation. The aggregation of the 41 food items into 12 food groups was used for descriptive presentation and substantive interpretation of the results; the three dietary patterns were identified statistically using principal component analysis.

Additionally, gender, age, area of residence, ethnocultural affiliation, shift in education, family income category (according to self-assessment of respondents/parents) and household food expenses were recorded.

Ethnic and cultural affiliation was determined by parents for children under the age of 14 and independently by older adolescents. Two groups have been identified for comparative analysis: Kazakh and non-Kazakh ethnicity. Participants from other ethnic groups were not included in the comparative analysis due to their small numbers.

### 2.5. Statistical Analysis

Statistical analyses were conducted using IBM SPSS Statistics (Version:27.0). The study sample was divided into three age groups: 7–10 years, 11–14 years, and 15–17 years. Descriptive statistics were used to summarize the characteristics of the study population. Categorical variables were expressed as frequencies and percentages.

Dietary intake was assessed using a food frequency questionnaire including four response options: “every day”, “several times a week”, “several times a month”, and “rarely or never”. For descriptive analyses, the full four-category distribution was presented (Appendix A). For the main comparative table, frequent consumption was presented, defined as intake every day or several times a week.

Differences in food consumption frequencies between age groups were evaluated using Pearson’s chi-square test. The magnitude of associations was estimated using Cramér’s V. Cramér’s V values were interpreted as follows: 0.10–0.19, weak association; 0.20–0.39, moderate association; 0.40–0.59, strong association; and ≥0.60, very strong association. A *p*-value < 0.05 was considered statistically significant.

To identify dietary patterns among schoolchildren, we applied Principal Component Analysis (PCA) [24]. For PCA and regression analysis, consumption frequency categories were converted to a numeric scale from 1 to 4, where 1 corresponded to the “rarely or never” category and 4 to the “daily” category. Higher values reflected higher frequency of consumption of the corresponding product. Prior to the analysis, variables were standardized (z-transformation) to eliminate the influence of differences in measurement scales. Component extraction was performed using the eigenvalue > 1 criterion, as well as visual inspection of the scree plot and factor interpretability. To improve interpretability, orthogonal varimax rotation was applied. Factor loadings ≥ 0.30 in absolute value were considered significant and were used to interpret the identified dietary patterns. For each participant, factor scores were calculated for each of the identified dietary patterns. The obtained values were used in subsequent analyses to assess differences between groups and to examine associations with sociodemographic characteristics (age, gender, socioeconomic indicators) using regression models.

## 3. Results

### 3.1. Sociodemographic Characteristics of the Sample

A total of 864 schoolchildren were included, of whom 47.9% were boys and 52.1% were girls (Table 1). The distribution by sex was generally similar across age groups. Overall, 55.2% of participants were of Kazakh ethnicity, while 44.7% were of non-Kazakh ethnicity, with only minor variation between age groups. Schools were located in two districts, with 53.9% of participants attending schools in the South-East district and 46.1% in the Maikuduk district.

Morning-shift attendance increased substantially with age, from 40.3% in children aged 7–10 years to 80.3% in those aged 11–14 years and 95.5% in adolescents aged 15–17 years, whereas afternoon-shift attendance showed the opposite pattern.

Family income also varied across age groups. Overall, 12.6% of participants reported a family income below USD 620 per month, 20.0% reported USD 620–950, 17.2% reported USD 950–1200, and 22.3% reported more than USD 1200, while income was unknown for 27.8% of respondents. The proportion of participants with unknown family income was higher in the older age groups. Monthly food expenditure above USD 430 was reported by 28.4% overall, with the highest proportion observed in the youngest age group (34.7%).

Regarding parental education, 78.1% of mothers had higher education, compared with 22% who had secondary education only. For fathers, 59.0% had higher education and 31% had secondary education, with some variation across age groups.

### 3.2. Age-Specific Patterns of Frequent Food Consumption

Among dairy products, frequent milk consumption was reported by 49.7% of children aged 7–10 years, 47.2% of those aged 11–14 years, and 34.1% of adolescents aged 15–17 years. An even more pronounced pattern was observed for fermented dairy products, with frequent consumption reported by 81.6%, 79.3%, and 23.2% of participants in the three age groups, respectively. Frequent consumption of cottage cheese, irimshik, and cheese also declined with age, from 55.2% in the youngest group to 23.2% in the oldest group (Table 2).

For fat-containing products, frequent consumption of butter and vegetable oil was high across all age groups, exceeding 86% in each group, and no significant age-related differences were observed. In contrast, frequent consumption of cooking fat/margarine remained relatively low, ranging from 12.0% to 20.1%, although the difference across age groups was statistically significant.

Within the meat group, frequent fresh meat consumption was consistently high in all age groups, ranging from 88.8% to 91.0%. However, age-related differences were observed for several processed meat products. Frequent sausage/processed meat consumption increased from 75.7% in children aged 7–10 years to 86.5% in adolescents aged 15–17 years. Frequent consumption of canned meat also rose markedly with age, from 13.9% in the youngest group to 53.6% in the oldest group. Similar differences were observed for offal, traditional meat products, and dried/smoked meat.

Frequent fresh fish consumption was low overall, reported by 15.6% of children aged 7–10 years, 9.7% of those aged 11–14 years, and 8.6% of those aged 15–17 years. Frequent egg consumption was more common, reported by 75.7%, 82.5%, and 69.3% of participants in the three age groups, respectively.

Among grain products, frequent consumption of bread products was universal in all age groups. Frequent consumption of pasta/homemade noodles remained high but declined slightly with age, from 90.3% in the youngest group to 80.9% in the oldest group. In contrast, frequent cereal consumption increased with age, from 75.3% to 89.5%.

Significant age-related differences were also observed for confectionery products. Frequent sugar consumption was reported by 90.6% of children aged 7–10 years, 95.1% of those aged 11–14 years, and 77.2% of adolescents aged 15–17 years. Frequent consumption of candies/chocolate/confectionery ranged from 72.2% to 86.5%, while honey was consumed frequently by 14.6% to 26.6% of participants depending on age group.

In the legumes, nuts, vegetables, and fruit categories, several age-related differences were also evident. Frequent legume consumption was highest in the 11–14-year group (39.5%), whereas frequent nut consumption increased sharply with age, from 13.9% in the youngest group to 61.4% in the oldest group. Frequent consumption of fresh vegetables remained high in all age groups, ranging from 85.8% to 95.1%. Frequent canned vegetable consumption, however, increased substantially with age, from 3.8% in the youngest group to 33.0% in the oldest group. Frequent fresh fruit consumption was high across all age groups, exceeding 86%, whereas frequent berry consumption remained low overall.

In the beverage category, clear age-related differences were observed. Frequent vegetable/fruit juice consumption declined with age, from 66.3% in children aged 7–10 years to 31.1% in those aged 15–17 years. In contrast, frequent coffee consumption rose dramatically, from 0% in the youngest group to 56.0% in the 11–14-year group and 88.8% in the oldest group. Frequent tea consumption was high in all age groups, exceeding 93%, whereas frequent consumption of sweetened carbonated beverages was common but did not differ significantly across groups.

The most pronounced age-related difference was observed for fast-food consumption. Frequent fast-food intake was reported by only 10.4% of children aged 7–10 years, compared with 66.7% of those aged 11–14 years and 87.6% of adolescents aged 15–17 years.

### 3.3. Principal Component Analysis

**Figure 1:**
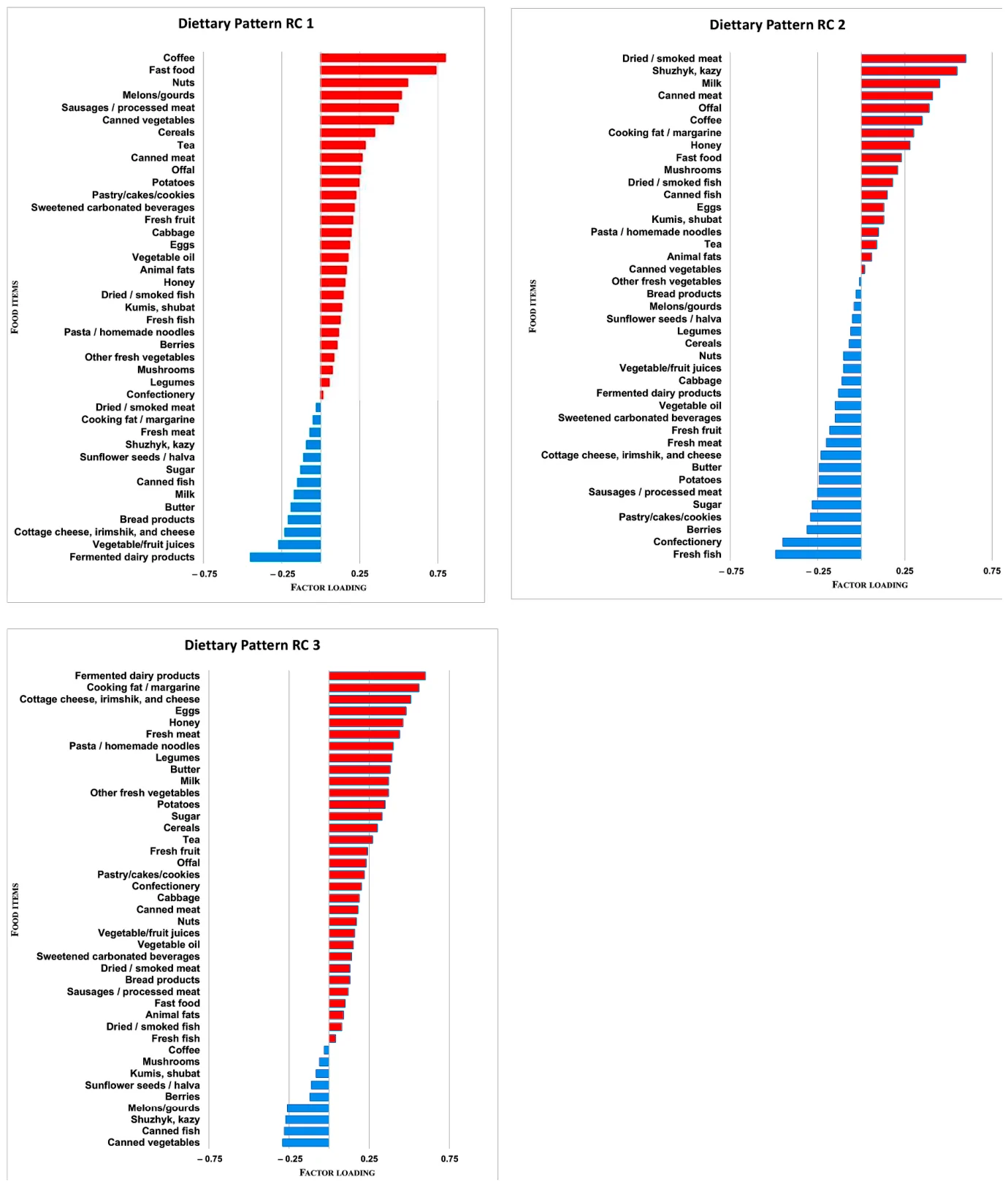
Factor loadings for dietary patterns identified by PCA. Total explained variance: 33.5%.

Figure 1 presents factor loadings for three dietary patterns identified using principal component analysis. Only factor loadings ≥ 0.30 were included in the analysis, allowing focus on the most meaningful associations between food groups and components.

The first dietary pattern (RC1) was interpreted as the “Processed and Fast Food Pattern.” The highest factor loadings were observed for coffee (Cramér’s V = 0.74) and fast food (Cramér’s V = 0.67), suggesting that they are key components of this pattern. Additional contributions were made by processed meat products, canned meat, organ meats, nuts, melons, potatoes, cereals, canned vegetables, and tea. The negative loading for juices indicates an inverse relationship with this pattern. Thus, RC1 characterizes a diet dominated by industrially processed and high-calorie foods.

The second dietary pattern (RC2), interpreted as the «Traditional meat-based pattern» is characterized by high consumption of traditional meat products (Cramér’s V = 0.64) (including shuzhyk and kazy), smoked and dried meat (Cramér’s V = 0.69), canned meat, and offal. A moderate positive association with coffee consumption is also observed. At the same time, negative associations are found with fresh fish (Cramér’s V = −0.51) and sweets (Cramér’s V = −0.44). This pattern reflects traditional dietary habits with a strong emphasis on animal-based products typical of the region.

The third dietary pattern (RC3), labeled as the «Mixed dairy-based pattern» is characterized by higher consumption of fermented dairy products (Cramér’s V = 0.55), margarine (Cramér’s V = 0.50), cheese (Cramér’s V = 0.49), as well as milk, butter, fresh meat, eggs, legumes, noodles, and honey. This pattern reflects a more diverse diet including both animal- and plant-based foods and may be considered a more balanced dietary pattern compared to the others identified.

**Figure 2:**
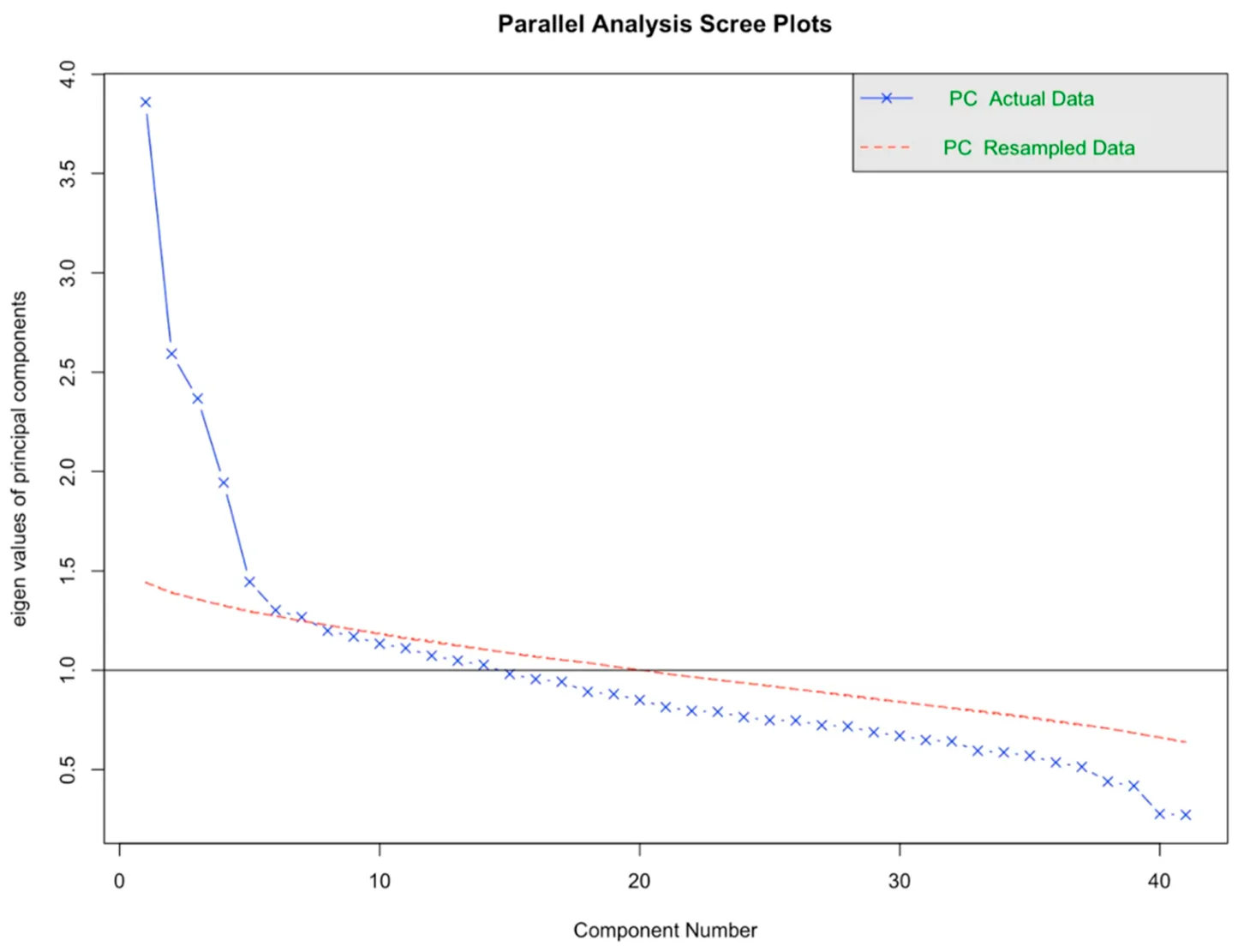
Scree plot analysis.

Figure 2 presents a parallel analysis scree plot illustrating the relationship between eigenvalues and component number. The blue line with markers represents eigenvalues derived from the observed data, while the red dashed lines indicate corresponding values obtained from randomly generated and permuted datasets. The horizontal line at the level of 1 denotes the classical Kaiser criterion for component retention. The first few components account for the majority of the variance and contain meaningful information, whereas subsequent components largely reflect random noise. Additionally, a clear “elbow” in the curve is observed after the first components, further supporting the appropriateness of limiting the number of factors in the model.

Table 3 shows the results of three regression models in which the established dietary patterns were assigned as the dependent variable. The analysis revealed that the first pattern, RC1, characterized by frequent consumption of processed foods and fast food, was favored by older adolescents (b = 1.18, *p* < 0.001), children of non-Kazakh ethnicity (b = 0.39, *p* < 0.001), and students in schools located in the “South-East” district (b = 0.16, *p*-value < 0.001). Factors associated with rejection of this dietary pattern include: children in a younger age group and monthly food expenditures of less than $200.

Adherence to the RC2 pattern, characterized by a predominantly national meat-based diet, was not detected. However, denial of this eating pattern was typical among younger children (b = −0.79), non-Kazakh (b = −1.23), and students in schools located in the “South-East” district (b = −0.49), who spend more than $380 per month on food (b = −0.31).

Adherence to the RC3 pattern, characterized by a mixed dairy-based diet, was demonstrated by those study participants who spend at least $260 per month on food (b = 0.55–0.59). This eating pattern was rejected by younger (b = −0.43) and older children (b = −0.62), and students in schools located in the “South-East” district (b = −0.27).

## 4. Discussion

There were three dietary patterns among schoolchildren we identified in our study. The first dietary pattern, RC1, was defined as the “processed and fast-food dietary pattern”. The second pattern, RC2, reflected a more traditional meat-based dietary pattern, while the third pattern, RC3, represented a mixed diet. Taken together, these patterns reflect the dietary behavior of school-age children and, more broadly, the current food culture of the population in Kazakhstan [20,25,26]. Our findings show the coexistence of dietary patterns rooted in traditional national food preferences and patterns shaped by the growing consumption of processed foods. This is consistent with the concept of the nutrition transition, which is typical for countries undergoing economic change, urbanization, and lifestyle transformation. Similar processes have been described in Latin America, Eastern Europe, the Middle East, and North Africa, where traditional foods remain part of everyday diets, while processed foods associated with Western dietary patterns become more common [27,28,29,30,31].

### 4.1. RC1: Processed and Fast-Food Dietary Pattern

The RC1 pattern in our study was mostly characterized by frequent consumption of fast food and processed products, which are usually energy-dense and low in nutritional value. This pattern included canned meat and offal, canned vegetables, processed meat products such as sausages and similar products, and cereal-based products. In terms of its food composition, RC1 is close to the Western dietary pattern described in studies from Europe and other regions [32,33,34,35,36,37]. Our findings are consistent with international evidence showing that urbanization contributes to changes in lifestyle and dietary behavior in countries undergoing the nutrition transition [28,29,30,38,39,40]. Moreover, a study by Kazakhstani researchers has reported a shift in food habits among young people toward greater consumption of processed foods [26]. Also, similar to our findings, studies from Europe and Asia have reported that adolescents and young people increasingly prefer caffeinated drinks, carbonated beverages, and sugar-sweetened beverages, while consumption of traditional beverages and natural fruit juices has declined [40,41,42,43,44]. Together with our earlier work on schoolchildren’s diets [5], the composition of RC1 suggests that this pattern may reflect broader changes in food behavior among urban children and adolescents.

Among the sociodemographic factors associated with adherence to the RC1 dietary pattern, age, ethnicity, residential district, and monthly household food expenditure showed the strongest associations. The clearest association was observed among older adolescents, suggesting that unhealthy food choices become more common with age. Younger children were less likely to adhere to the RC1 pattern, which may partly reflect the provision of free school meals in the lower grades. Similar age-related patterns have been reported in previous studies [45,46,47,48]. Greater autonomy in food choices during adolescence, reduced parental supervision, more time spent outside the home, easier access to fast food, the expansion of food delivery services, and exposure to digital media may all contribute to this pattern.

There were no clear gender differences in the RC1 pattern. Evidence on gender differences in adolescent dietary patterns remains inconsistent. Feraco et al. (2024) and Mekonen et al. (2025) reported that boys were more likely to consume red and processed meat, whereas girls more often followed healthier dietary patterns [49,50]. In contrast, other studies, including systematic reviews, suggest that adolescents’ food choices are influenced more strongly by social and cultural factors than by gender alone [48,51,52,53].

The analysis by ethnicity showed that children of non-Kazakh ethnicity were more likely to adhere to the RC1 dietary pattern than Kazakh children. This finding is consistent with studies from Kazakhstan indicating that traditional Kazakh cuisine remains an important cultural value despite ongoing urbanization and modernization [54,55,56]. Similar differences across ethnic and cultural groups have also been reported among European adolescents [57,58,59].

Higher adherence to RC1 was observed among pupils attending schools in the South-East district than among those in Maikuduk. This difference may reflect characteristics of the local food environment. Compared with Maikuduk, the South-East district is more urbanized and has greater availability of shopping centres, fast-food outlets, and vending machines selling snacks and beverages, which may contribute to less healthy food choices. Similar associations between the food environment and unhealthy dietary patterns have been reported internationally [60,61,62,63] and in Kazakhstan, where urbanization and greater access to fast food have been linked to less healthy eating habits [12,64,65].

No clear differences in RC1 adherence were found between pupils attending morning and afternoon school shifts. Although many studies have linked breakfast skipping with higher consumption of sugar-sweetened beverages, unhealthy foods, and poorer sleep patterns [66,67,68,69,70], other evidence suggests that the influence of school schedules and meal timing may be outweighed by broader socioeconomic factors [66,71]. In our study, household income was not associated with RC1, whereas monthly household food expenditure showed a clearer relationship. Lower spending on food was associated with lower adherence to the RC1 pattern. One possible explanation is that limited food budgets reduce the purchase of fast food, ready-to-eat snacks, and other foods characteristic of this pattern. Another possibility is that some families, regardless of income, prioritize home-cooked meals and staple foods over eating outside the home. Previous studies have shown that lower socioeconomic status, often measured by household income, is associated with poorer diet quality and greater reliance on energy-dense, nutrient-poor foods. Financial constraints also influence household food choices and adolescents’ dietary behaviours [2,48,72,73,74,75].

Parental education was not associated with RC1 in the present study. This contrasts with findings from Europe, Asia, and the Americas, where higher parental education, particularly maternal education, has been linked to healthier food choices, greater nutrition knowledge, and better diet quality among children and adolescents [76,77,78,79,80,81].

### 4.2. RC2: Traditional Meat-Based Pattern

The second dietary pattern is defined by positive loadings on traditional Kazakh preserved meat preparations, specifically shuzhyk and kazy, smoked and dried meat, canned meat, and offal, with a moderate positive loading for coffee. Fresh fish and sweets load negatively on this component.

The foods carrying the highest positive loadings in RC2 match those long identified in the ethnographic record of Kazakh food culture as central to the regional diet [82]. Their persistence in the diets of urban schoolchildren, generations after the pastoral economy that produced them, points to strong intergenerational transmission of food preference within Kazakh households, consistent with the continued role of livestock as the structural foundation of the national food economy [83,84]. A cross-sectional study of adults in Western Kazakhstan reported a comparable generational gradient, with markedly lower adherence to a traditional dietary pattern among younger adults than among those aged 60 to 65 [26], a finding that parallels the age patterning observed in the present child sample and suggests that the retreat from traditional eating in Kazakhstan is not confined to any single cohort.

Generational erosion of a traditional diet is not, by itself, evidence of nutritional improvement. The comparative literature on traditional dietary patterns indicates that protective associations with obesity and chronic disease concentrate in patterns rich in plant-based foods, dietary fiber, and fish, rather than in those built primarily around preserved animal products [85]. RC2, as captured in the present factor structure, corresponds to the latter profile: it is centered on cured and preserved meat and carries no positive loading from vegetables, fruit, or fish. The present study did not assess body weight, blood lipids, or other cardiometabolic markers, so this compositional profile cannot be read as evidence that children following RC2 already show adverse metabolic outcomes. It places RC2, structurally, apart from the dietary profiles that published cohorts associate with more favorable long-term health trajectories [86,87].

The negative loading for fresh fish reflects the traditionally low consumption of fish in Kazakhstan, where livestock has historically been the foundation of the national diet [82,83]. This pattern extends beyond childhood. A national survey of young adults reported that most participants consumed fish less than once per month, and average per capita fish consumption remains well below recommended levels [25,88].

Younger children in the present sample showed lower adherence to RC2 relative to the middle age group, while the oldest adolescents showed no significant deviation from the reference category. Lower adherence among younger children may reflect family food practices and age-related differences in food preferences, as younger children’s diets are more strongly influenced by parents than those of older adolescents [89,90].

Non-Kazakh ethnicity was the strongest predictor of lower adherence to RC2, with a substantially larger effect than observed for RC1. This likely reflects the ethnic specificity of RC2 foods, such as shuzhyk, kazy, smoked horsemeat, and offal, which are deeply rooted in Kazakh culinary traditions and are less commonly consumed by non-Kazakh households [82]. Similar ethnic differences in traditional dietary patterns have been reported in a systematic review of co-located ethnic groups, where traditional foods showed greater between-group variation than processed foods [91]. In contrast to findings from many countries, parental education was not associated with RC2 adherence. While higher parental education has often been linked to healthier dietary behaviours in children [92], our findings suggest that adherence to this dietary pattern is driven primarily by cultural identity rather than socioeconomic factors. This interpretation is consistent with evidence from the WHO European Childhood Obesity Surveillance Initiative, which found that children’s dietary habits in Kazakhstan were influenced by a combination of cultural and socioeconomic factors that did not follow the typical educational gradient observed in Europe [93].

Higher household food expenditure was associated with lower adherence to RC2. This may reflect greater dietary diversity among households with larger food budgets rather than lower consumption of traditional foods. As households expand their food choices, the relative contribution of traditional foods to the overall dietary pattern may decrease, resulting in lower RC2 scores [88].

### 4.3. RC3: Relatively Diverse Mixed Dietary Pattern

The RC3 dietary pattern was characterized by higher consumption of dairy products, fermented dairy products such as kefir and ayran, butter, fresh meat, legumes, eggs, and noodles. Compared with RC1 and RC2, this pattern was relatively more diverse, providing several sources of animal and plant protein. Even so, it cannot be considered fully balanced because fruits, vegetables, berries, and fish contributed little to this dietary pattern.

The high contribution of fermented dairy products is particularly relevant in the context of Central Asian dietary traditions. Fermented dairy beverages, including kefir, ayran, kumys, and shubat, have long been part of the traditional Kazakh diet and are valued for their nutritional qualities and probiotic properties [94,95]. Therefore, the presence of kefir, ayran, sour cream, and cream may be a favorable feature of RC3, as fermented dairy consumption has been associated with improved gastrointestinal and bone health, better weight maintenance, and a lower risk of several chronic diseases, including cardiovascular disease and some cancers [96]. Fermented dairy consumption also differed by ethnicity and residential district. Children of non-Kazakh ethnicity were less likely to consume these products, possibly reflecting stronger preservation of traditional dairy foods among Kazakh families. Lower consumption among pupils in the South-East district may reflect a more urbanized lifestyle, differences in food availability, or household food practices. Similar dietary shifts have been reported in Kazakhstan and other populations undergoing nutrition transition [26].

Another important feature of RC3 was the presence of fresh meat, while processed meat products, such as sausages and similar items, were not part of this model. Fresh meat, eggs, and legumes may also reflect a tendency toward home-prepared meals. This can be considered a favorable marker of the RC3 pattern, since home cooking has been associated with healthier dietary intake, including higher consumption of fruit and vegetables, as well as better weight-related outcomes [97]. Fruits, vegetables, berries, and fish contributed little to this pattern, indicating that it does not fully align with recommendations for a balanced diet. Overall, RC3 is better described as a relatively diverse dietary pattern than a balanced one. This interpretation is consistent with studies showing that greater dietary diversity among children and adolescents does not necessarily translate into adequate consumption of protective food groups. Adolescents frequently report low intakes of fruits and vegetables despite otherwise varied diets [98,99].

Adherence to RC3 declined with age, whereas adherence to RC1 increased among older adolescents. This shift suggests that dietary habits become less favorable during adolescence, possibly because of greater independence, more frequent eating outside the home, peer influences, and increased exposure to urban food environments. Similar trends have been reported in studies documenting low dietary diversity and inadequate consumption of nutrient-dense foods among adolescents. Jenkins et al. found that foods rich in key micronutrients, including vitamin A-rich fruits and vegetables and dark green vegetables, were consumed by only a minority of adolescents, indicating poor adherence to dietary recommendations [100]. Higher household food expenditure was also associated with greater adherence to RC3. This finding is consistent with evidence that healthier dietary patterns often cost more than less healthy alternatives. A meta-analysis by Rao et al. found that healthier foods and dietary patterns were more expensive than less healthy options when assessed by daily intake or energy-adjusted units [101]. In the present study, higher food expenditure may have enabled families to purchase a wider range of foods, including dairy products, fresh meat, eggs, and legumes. Even so, the limited contribution of fruits, vegetables, berries, and fish indicates that higher food spending alone does not guarantee a healthy diet. The decline in RC3 adherence with age may also reflect the transition from primary to secondary school, a period often accompanied by changes in daily routines, food access, autonomy, and meal structure. A systematic review by Peral-Suárez et al. found that diet quality tends to decline during this transition, with less frequent consumption of breakfast, fruits, vegetables, and milk as children progress through school [102]. These findings may help explain why the relatively diverse RC3 pattern was less common among older adolescents.

### 4.4. Practical Implications

The three dietary patterns identified in this study highlight several priorities for nutrition policy and practice in Kazakhstan. Since adherence to the processed and fast-food dietary pattern increased with age, preventive interventions should begin before adolescence, when dietary habits become more established. School-based programmes that combine nutrition education with improvements to the school food environment have shown greater effectiveness than education alone in promoting healthier eating behaviours among children and adolescents [103,104,105]. Given the higher adherence to RC1 among pupils attending schools in the more urbanized district, interventions should also address the local food environment by increasing the availability of healthier foods and limiting exposure to energy-dense snacks and sugar-sweetened beverages in and around schools.

The findings also suggest that nutrition promotion in Kazakhstan should build on existing dietary traditions rather than replace them. The traditional meat-based pattern reflected cultural continuity but included limited amounts of fruits, vegetables, and fish, whereas the relatively diverse mixed pattern (RC3) incorporated fermented dairy products, legumes, eggs, and fresh meat but still lacked several food groups recommended for a balanced diet. Nutrition education and school meal policies should encourage the preservation of culturally accepted foods with established nutritional value, such as fermented dairy products, while increasing access to fruits, vegetables, whole grains, and fish. The association between household food expenditure and dietary patterns also indicates that economic factors influence food choices. School meal programmes and other public health interventions may help improve access to nutrient-dense foods, particularly for children from households with more limited food budgets [101,103].

### 4.5. Limitations

The present study has several limitations. Firstly, the cross-sectional design allows us to identify only associations between the characteristics of schoolchildren, family, and eating habits. Secondly, the sample was limited to students of public schools in two districts of Karaganda, which limits the possibility of generalizing the results to all schoolchildren in Kazakhstan, especially rural areas, small towns and regions with a different socio-economic and ethnocultural structure. The cluster structure at the school level was taken into account in the sample size calculation using a design effect (DEFF) of 2.0. However, in the statistical analysis, individual observations were treated as independent, without additional adjustment for clustering at the school level. This should be taken into consideration when interpreting the results. In addition, the method used to assess the frequency of food consumption did not allow us to determine portion sizes, daily calorie intake and intake of macro- and micronutrients, so the results do not reflect the nutritional adequacy of the diet. Data based on students’ self-reports and parents’ responses may also be subject to recall and social desirability bias. This may have resulted in over-reporting of healthy food consumption and underreporting of less healthy foods, such as fast food, sugar-sweetened beverages, and snacks. In addition, parents may not have been fully aware of foods consumed by their children outside the home. Finally, the study did not assess the place of food consumption, family food practices, the availability of food outlets near schools, as well as differences in nutrition on weekdays, weekends, and holidays. Taking these factors into account in future studies will allow for a deeper interpretation of the eating behavior of schoolchildren. The questionnaire underwent pilot testing for feasibility and linguistic clarity; however, the absence of a formal reliability assessment, back-translation, and external validation is a limitation of the study.

## 5. Conclusions

The study showed that the nutrition of Karaganda schoolchildren increases with increasing age and the peculiarities of eating behavior, which may be associated with greater food independence and urban conditions. Frequent consumption of fast food, coffee, processed and canned meat products occupies a prominent place in the diet of high school students, while the frequency of consumption of milk and fermented dairy products is decreasing. Sugary carbonated drinks remain widespread in all age groups, while fresh fish and berries are rarely present in the diet of schoolchildren.

The relatively most favorable of the identified dietary patterns is not optimal, since vegetables, fruits, berries and fish are not sufficiently represented in it. The results of the study should be considered not as evidence of a lack of healthy foods in the diet, but as a sign of an unbalanced dietary choice, especially among adolescents.

The data obtained can be used to substantiate targeted preventive measures for older schoolchildren, whose food independence is increasing. When developing programs for schools and parents, it is advisable to take into account the identified age differences and focus on reducing the frequency of consumption of fast food, sugary carbonated drinks, coffee, and processed and canned meat products.

## Figures and Tables

**Table 1:** Socio-demographic characteristics of the respondents.

Variable	Total, *n* = 864 (%)	Children Age Group, *n* (%)		
		7–10 Years (*n* = 288)	11–14 Years (*n* = 309)	15–17 Years (*n* = 267)
Male	414 (47.92)	133 (46.18)	147 (47.57)	134 (50.2)
Female	450 (52.08)	155 (53.82)	162 (52.43)	133 (49.8)
Children of Kazakh ethnicity	477 (55.21)	159 (55.21)	179 (57.93)	139 (52.05)
Children of non-Kazakh ethnicity	387 (44.79)	129 (44.79)	130 (42.07)	128 (47.94)
School in “Maikuduk” district	398 (46.06)	136 (47.22)	132 (42.72)	130 (48.69)
School in “South” district	466 (53.94)	152 (52.78)	177 (57.28)	137 (51.31)
Morning shift	619 (71.64)	116 (40.28)	248 (80.26)	255 (95.51)
Afternoon shift	245 (28.36)	172 (59.72)	61 (19.74)	12 (4.49)
Family income less than 620$	109 (12.62)	52 (18.06)	40 (12.94)	17 (6.37)
Family income 620–950$	173 (20.02)	72 (25)	47 (15.21)	54 (20.23)
Family income less than 950–1200$	149 (17.25)	44 (15.28)	56 (18.12)	49 (18.35)
Family income more than 1200$	193 (22.34)	67 (23.26)	73 (23.62)	53 (19.85)
Family income unknown	240 (27.78)	53 (18.40)	93 (30.09)	94 (35.21)
Monthly food expenses less than 200$	27 (3.13)	7 (2.43)	12 (3.88)	8 (2.99)
Monthly food expenses 200–250$	129 (14.93)	38 (13.19)	54 (17.48)	37 (13.86)
Monthly food expenses 260–310$	122 (14.12)	37 (12.85)	43 (13.92)	42 (15.73)
Monthly food expenses 320–370$	138 (15.97)	42 (14.58)	51 (16.50)	45 (16.85)
Monthly food expenses 380–430$	140 (16.20)	44 (15.28)	56 (18.12)	40 (14.98)
Monthly food expenses more than 430$	264 (30.56)	100 (34.72)	80 (25.89)	84 (31.46)
Monthly food expenses unknown	44 (5.09)	20 (6.94)	13 (4.21)	11 (4.12)
Mother’s education—secondary school	189 (21.88)	58 (20.14)	71 (22.98)	60 (22.47)
Mother’s education—higher	675 (78.12)	230 (79.86)	238 (77.02)	207 (77.52)
Father’s education—secondary school	268 (31.02)	72 (25)	110 (35.59)	86 (9.95)
Father’s education—higher	510 (59.02)	200 (69.44)	170 (55.02)	140 (52.43)
Father’s education—missed	86 (9.95)	16 (5.56)	29 (9.38)	41 (15.36)

**Table 2:** Frequent consumption of selected food items across age groups.

Product		7–10 Years (*n* = 288)	11–14 Years (*n* = 309)	15–17 Years (*n* = 267)	*p*-Value	Cramér’s V
Milk and dairy products	Milk	143 (49.7%)	146 (47.2%)	91 (34.1%)	<0.001	0.135
	Fermented dairy products	235 (81.6%)	245 (79.3%)	62 (23.2%)	<0.001	0.547
	Cottage cheese, irimshik, and cheese	159 (55.2%)	117 (37.9%)	62 (23.2%)	<0.001	0.263
High-fat foods (>50% fat content)	Butter	262 (91.0%)	275 (89.0%)	231 (86.5%)	0.248	0.057
	Vegetable oil	272 (94.4%)	302 (97.7%)	257 (96.3%)	0.111	0.071
	Cooking fat/margarine	36 (12.5%)	62 (20.1%)	32 (12.0%)	0.009	0.105
	Animal fats	18 (6.3%)	29 (9.4%)	16 (6.0%)	0.209	0.060
Meat and meat products	Fresh meat	262 (91.0%)	278 (90.0%)	237 (88.8%)	0.688	0.029
	Sausages/processed meat	218 (75.7%)	227 (73.5%)	231 (86.5%)	<0.001	0.136
	Offal	97 (33.7%)	101 (32.7%)	144 (53.9%)	<0.001	0.196
	Traditional meat products (shuzhyk, kazy)	27 (9.4%)	111 (35.9%)	77 (28.8%)	<0.001	0.262
	Dried/smoked meat	28 (9.7%)	96 (31.1%)	82 (30.7%)	<0.001	0.234
	Canned meat	40 (13.9%)	120 (38.8%)	143 (53.6%)	<0.001	0.338
Fish	Fresh fish	45 (15.6%)	30 (9.7%)	23 (8.6%)	0.018	0.097
Eggs	Eggs	218 (75.7%)	255 (82.5%)	185 (69.3%)	0.001	0.127
Grains and grain products	Bread products	288 (100.0%)	309 (100.0%)	267 (100.0%)	NA	NA
	Pasta/homemade noodles	260 (90.3%)	267 (86.4%)	216 (80.9%)	0.006	0.109
	Cereals	217 (75.3%)	250 (80.9%)	239 (89.5%)	<0.001	0.148
Confectionery products	Pastry/cakes/cookies	221 (76.7%)	233 (75.4%)	235 (88.0%)	<0.001	0.138
	Sugar	261 (90.6%)	294 (95.1%)	206 (77.2%)	<0.001	0.233
	Confectionery	249 (86.5%)	223 (72.2%)	213 (79.8%)	<0.001	0.147
	Honey	42 (14.6%)	65 (21.0%)	71 (26.6%)	0.002	0.119
	Sunflower seeds/halva	16 (5.6%)	0 (0.0%)	0 (0.0%)	<0.001	0.194
Legumes and nuts	Legumes	40 (13.9%)	122 (39.5%)	27 (10.1%)	<0.001	0.320
	Nuts	40 (13.9%)	77 (24.9%)	164 (61.4%)	<0.001	0.424
Vegetables and processed vegetable products	Potatoes	248 (86.1%)	223 (72.2%)	237 (88.8%)	<0.001	0.192
	Cabbage	99 (34.4%)	155 (50.2%)	135 (50.6%)	<0.001	0.151
	Other fresh vegetables	247 (85.8%)	294 (95.1%)	248 (92.9%)	<0.001	0.143
	Canned vegetables	11 (3.8%)	69 (22.3%)	88 (33.0%)	<0.001	0.300
Fruits, berries, and processed fruit products	Melons/gourds	0 (0.0%)	0 (0.0%)	45 (16.9%)	<0.001	0.351
	Fresh fruit	256 (88.9%)	266 (86.1%)	250 (93.6%)	0.013	0.100
	Berries	0 (0.0%)	17 (5.5%)	28 (10.5%)	<0.001	0.189
Beverages	Vegetable/fruit juices	191 (66.3%)	170 (55.0%)	83 (31.1%)	<0.001	0.287
	Tea	270 (93.8%)	292 (94.5%)	257 (96.3%)	0.398	0.046
	Coffee	0 (0.0%)	173 (56.0%)	237 (88.8%)	<0.001	0.723
	Sweetened carbonated beverages	203 (70.5%)	210 (68.0%)	196 (73.4%)	0.360	0.049
Fast food	Fast food	30 (10.4%)	206 (66.7%)	234 (87.6%)	<0.001	0.648

**Table 3:** Sociodemographic factors determining dietary patterns of schoolchildren.

Independent Variables	RC1	RC2	RC3
7–10 age group	−0.76 ***	−0.79 ***	−0.43 ***
11–14 age group	Ref.	Ref.	Ref.
15–17 age group	1.18 ***	−0.08	−0.62 ***
Male	−0.03	−0.04	0.09
Female	Ref.	Ref.	Ref.
Children of Kazakh ethnicity	Ref.	Ref.	Ref.
Children of non-Kazakh ethnicity	0.39 ***	−1.23 ***	−0.17
School in “Maikuduk” district	Ref.	Ref.	Ref.
School in “South-East” district	0.16 ***	−0.49 ***	−0.27 ***
Morning shift	Ref.	Ref.	Ref.
Afternoon shift	−0.06	−0.09	0.18
Family income less than 620$	−0.05	0.00	−0.09
Family income 620–950$	Ref.	Ref.	Ref.
Family income 950–1200$	−0.07	0.00	0.03
Family income more than 1200$	−0.08	−0.02	0.01
Family income unknown	−0.07	0.03	−0.07
Monthly food expenses less than 200$	−0.3 *	0.23	0.28
Monthly food expenses 200–250$	Ref.	Ref.	Ref.
Monthly food expenses 260–310$	0.03	−0.2	0.55 **
Monthly food expenses 320–370$	−0.03	−0.10	0.56 ***
Monthly food expenses 380–430$	0.12	−0.31 ***	0.59 ***
Monthly food expenses more than 430$	−0.01	−0.22 **	0.55 ***
Monthly food expenses unknown	0.25	−0.21	0.33 *
Mother’s education—secondary school	−0.05	0.06	−0.09
Mother’s education—higher	Ref.	Ref.	Ref.
Father’s education—secondary school	0.01	0.03	0.21
Father’s education—higher	0.00	−0.07	0.13

* For all models: (* *p* < 0.05, ** *p* < 0.01, *** *p* < 0.001 VIF: 1.01–1.19).

## Data Availability

The data presented in this study are available on reasonable request from the corresponding authors due to privacy and ethical reasons.

## Appendix A. Frequency of Consumption of Food Items by Age Group

**Product**	**Frequency of Consumption**	**7–10 Years (*n* = 288)**	**11–14 Years (*n* = 309)**	**15–17 Years (*n* = 267)**	**χ^2^ (*df*)**	***p***	**V Cramer**
Milk	Every day	70 (24.3%)	110 (35.6%)	52 (19.5%)	79,239 (6)	<0.001	0.214
	Several times a week	73 (25.3%)	36 (11.7%)	39 (14.6%)			
	Several times a month	52 (18.1%)	102 (33.0%)	123 (46.1%)			
	Rarely or never	93 (32.3%)	61 (19.7%)	53 (19.9%)			
Kefir, ayran, yogurt, sour cream, cream	Every day	69 (24.0%)	141 (45.6%)	22 (8.2%)	324,486 (6)	<0.001	0.433
	Several times a week	166 (57.6%)	104 (33.7%)	40 (15.0%)			
	Several times a month	46 (16.0%)	64 (20.7%)	163 (61.0%)			
	Rarely or never	7 (2.4%)	0 (0.0%)	42 (15.7%)			
Cottage cheese, irimshik, cheese	Every day	56 (19.4%)	51 (16.5%)	22 (8.2%)	66,972 (6)	<0.001	0.197
	Several times a week	103 (35.8%)	66 (21.4%)	40 (15.0%)			
	Several times a month	115 (39.9%)	155 (50.2%)	163 (61.0%)			
	Rarely or never	14 (4.9%)	37 (12.0%)	42 (15.7%)			
Kumys, shubat	Several times a month	1 (0.3%)	1 (0.3%)	14 (5.2%)	24,456 (2)	<0.001	0.168
	Rarely or never	287 (99.7%)	308 (99.7%)	253 (94.8%)			
Butter	Every day	195 (67.7%)	161 (52.1%)	87 (32.6%)	94,085 (6)	<0.001	0.233
	Several times a week	67 (23.3%)	114 (36.9%)	144 (53.9%)			
	Several times a month	24 (8.3%)	34 (11.0%)	22 (8.2%)			
	Rarely or never	2 (0.7%)	0 (0.0%)	14 (5.2%)			
Vegetable oil	Every day	194 (67.4%)	128 (41.4%)	209 (78.3%)	110,466 (6)	<0.001	0.253
	Several times a week	78 (27.1%)	174 (56.3%)	48 (18.0%)			
	Several times a month	8 (2.8%)	7 (2.3%)	5 (1.9%)			
	Rarely or never	8 (2.8%)	0 (0.0%)	5 (1.9%)			
Cooking fat, margarine	Every day	3 (1.0%)	19 (6.1%)	9 (3.4%)	21,086 (6)	0.002	0.110
	Several times a week	33 (11.5%)	43 (13.9%)	23 (8.6%)			
	Several times a month	30 (10.4%)	45 (14.6%)	28 (10.5%)			
	Rarely or never	222 (77.1%)	202 (65.4%)	207 (77.5%)			
Animal fats	Several times a week	18 (6.3%)	29 (9.4%)	16 (6.0%)	107,419 (4)	<0.001	0.249
	Several times a month	39 (13.5%)	155 (50.2%)	113 (42.3%)			
	Rarely or never	231 (80.2%)	125 (40.5%)	138 (51.7%)			
Fresh meat	Every day	167 (58.0%)	201 (65.0%)	125 (46.8%)	32,760 (6)	<0.001	0.138
	Several times a week	95 (33.0%)	77 (24.9%)	112 (41.9%)			
	Several times a month	15 (5.2%)	26 (8.4%)	28 (10.5%)			
	Rarely or never	11 (3.8%)	5 (1.6%)	2 (0.7%)			
Sausages/processed meat	Every day	52 (18.1%)	136 (44.0%)	169 (63.3%)	144,738 (6)	<0.001	0.289
	Several times a week	166 (57.6%)	91 (29.4%)	62 (23.2%)			
	Several times a month	61 (21.2%)	54 (17.5%)	28 (10.5%)			
	Rarely or never	9 (3.1%)	28 (9.1%)	8 (3.0%)			
Offal	Every day	17 (5.9%)	54 (17.5%)	27 (10.1%)	256,729 (6)	<0.001	0.385
	Several times a week	80 (27.8%)	47 (15.2%)	117 (43.8%)			
	Several times a month	17 (5.9%)	114 (36.9%)	106 (39.7%)			
	Rarely or never	174 (60.4%)	94 (30.4%)	17 (6.4%)			
Traditional meat products (shuzhyk, kazy)	Every day	27 (9.4%)	111 (35.9%)	77 (28.8%)	61,143 (4)	<0.001	0.188
	Several times a week	132 (45.8%)	88 (28.5%)	91 (34.1%)			
	Several times a month	129 (44.8%)	110 (35.6%)	99 (37.1%)			
Dried/smoked meat	Rarely or never	28 (9.7%)	96 (31.1%)	82 (30.7%)	53,498 (4)	<0.001	0.176
	Every day	131 (45.5%)	131 (42.4%)	99 (37.1%)			
	Several times a week	129 (44.8%)	82 (26.5%)	86 (32.2%)			
Canned meat	Every day	0 (0.0%)	104 (33.7%)	118 (44.2%)	260,607 (6)	<0.001	0.388
	Several times a week	40 (13.9%)	16 (5.2%)	25 (9.4%)			
	Several times a month	77 (26.7%)	65 (21.0%)	108 (40.4%)			
	Rarely or never	171 (59.4%)	124 (40.1%)	16 (6.0%)			
Canned fish	Several times a month	20 (6.9%)	5 (1.6%)	3 (1.1%)	19,010 (2)	<0.001	0.148
	Rarely or never	268 (93.1%)	304 (98.4%)	264 (98.9%)			
Fresh fish	Several times a week	45 (15.6%)	30 (9.7%)	23 (8.6%)	13,840 (4)	0.008	0.089
	Several times a month	90 (31.3%)	92 (29.8%)	105 (39.3%)			
	Rarely or never	153 (53.1%)	187 (60.5%)	139 (52.1%)			
Dried/smoked fish	Several times a month	0 (0.0%)	45 (14.6%)	34 (12.7%)	44,052 (2)	<0.001	0.226
	Rarely or never	288 (100.0%)	264 (85.4%)	233 (87.3%)			
Eggs	Every day	22 (7.6%)	48 (15.5%)	104 (39.0%)	136,740 (6)	<0.001	0.281
	Several times a week	196 (68.1%)	207 (67.0%)	81 (30.3%)			
	Several times a month	55 (19.1%)	52 (16.8%)	73 (27.3%)			
	Rarely or never	15 (5.2%)	2 (0.6%)	9 (3.4%)			
Bread products	Every day	288 (100.0%)	309 (100.0%)	256 (95.9%)	24,913 (2)	<0.001	0.170
	Several times a week	0 (0.0%)	0 (0.0%)	11 (4.1%)			
Pasta, homemade noodles	Every day	39 (13.5%)	44 (14.2%)	106 (39.7%)	125,800 (6)	<0.001	0.270
	Several times a week	221 (76.7%)	223 (72.2%)	110 (41.2%)			
	Several times a month	17 (5.9%)	42 (13.6%)	27 (10.1%)			
	Rarely or never	11 (3.8%)	0 (0.0%)	24 (9.0%)			
Cereals (rice, millet, buckwheat, rolled oats, etc.)	Every day	54 (18.8%)	98 (31.7%)	153 (57.3%)	116,940 (6)	<0.001	0.260
	Several times a week	163 (56.6%)	152 (49.2%)	86 (32.2%)			
	Several times a month	57 (19.8%)	59 (19.1%)	15 (5.6%)			
	Rarely or never	14 (4.9%)	0 (0.0%)	13 (4.9%)			
Pastry products (cakes, pastries, cookies)	Every day	51 (17.7%)	107 (34.6%)	96 (36.0%)	51,238 (6)	<0.001	0.172
	Several times a week	170 (59.0%)	126 (40.8%)	139 (52.1%)			
	Several times a month	65 (22.6%)	66 (21.4%)	25 (9.4%)			
	Rarely or never	2 (0.7%)	10 (3.2%)	7 (2.6%)			
Sugar	Every day	186 (64.6%)	177 (57.3%)	158 (59.2%)	104,285 (6)	<0.001	0.246
	Several times a week	75 (26.0%)	117 (37.9%)	48 (18.0%)			
	Several times a month	27 (9.4%)	15 (4.9%)	27 (10.1%)			
	Rarely or never	0 (0.0%)	0 (0.0%)	34 (12.7%)			
Confectionery (candies, chocolate, marmalade, etc.)	Every day	194 (67.4%)	114 (36.9%)	123 (46.1%)	67,017 (6)	<0.001	0.197
	Several times a week	55 (19.1%)	109 (35.3%)	90 (33.7%)			
	Several times a month	22 (7.6%)	70 (22.7%)	43 (16.1%)			
	Rarely or never	17 (5.9%)	16 (5.2%)	11 (4.1%)			
Honey	Every day	11 (3.8%)	13 (4.2%)	29 (10.9%)	55,831 (6)	<0.001	0.180
	Several times a week	31 (10.8%)	52 (16.8%)	42 (15.7%)			
	Several times a month	122 (42.4%)	183 (59.2%)	115 (43.1%)			
	Rarely or never	124 (43.1%)	61 (19.7%)	81 (30.3%)			
Sunflower seeds, halva	Several times a week	16 (5.6%)	0 (0.0%)	0 (0.0%)	48,842 (4)	<0.001	0.168
	Several times a month	130 (45.1%)	119 (38.5%)	147 (55.1%)			
	Rarely or never	142 (49.3%)	190 (61.5%)	120 (44.9%)			
Legumes (beans, peas, etc.)	Several times a week	40 (13.9%)	122 (39.5%)	27 (10.1%)	94,172 (4)	<0.001	0.233
	Several times a month	156 (54.2%)	97 (31.4%)	150 (56.2%)			
	Rarely or never	92 (31.9%)	90 (29.1%)	90 (33.7%)			
Nuts	Every day	0 (0.0%)	21 (6.8%)	42 (15.7%)	239,831 (6)	<0.001	0.373
	Several times a week	40 (13.9%)	56 (18.1%)	122 (45.7%)			
	Several times a month	223 (77.4%)	138 (44.7%)	88 (33.0%)			
	Rarely or never	25 (8.7%)	94 (30.4%)	15 (5.6%)			
Potatoes	Every day	65 (22.6%)	116 (37.5%)	109 (40.8%)	67,797 (4)	<0.001	0.198
	Several times a week	183 (63.5%)	107 (34.6%)	128 (47.9%)			
	Several times a month	40 (13.9%)	86 (27.8%)	30 (11.2%)			
Cabbage (fresh, sauerkraut)	Several times a week	99 (34.4%)	155 (50.2%)	135 (50.6%)	26,899 (4)	<0.001	0.125
	Several times a month	156 (54.2%)	142 (46.0%)	114 (42.7%)			
	Rarely or never	33 (11.5%)	12 (3.9%)	18 (6.7%)			
Other fresh vegetables	Every day	117 (40.6%)	150 (48.5%)	88 (33.0%)	42,786 (6)	<0.001	0.157
	Several times a week	130 (45.1%)	144 (46.6%)	160 (59.9%)			
	Several times a month	31 (10.8%)	15 (4.9%)	19 (7.1%)			
	Rarely or never	10 (3.5%)	0 (0.0%)	0 (0.0%)			
Canned vegetables	Several times a week	11 (3.8%)	69 (22.3%)	88 (33.0%)	93,645 (4)	<0.001	0.233
	Several times a month	193 (67.0%)	155 (50.2%)	149 (55.8%)			
	Rarely or never	84 (29.2%)	85 (27.5%)	30 (11.2%)			
Mushrooms	Several times a month	69 (24.0%)	148 (47.9%)	105 (39.3%)	37,236 (2)	<0.001	0.208
	Rarely or never	219 (76.0%)	161 (52.1%)	162 (60.7%)			
Melons/gourds (watermelon, melon, pumpkin, zucchini, pattypan squash)	Several times a week	0 (0.0%)	0 (0.0%)	45 (16.9%)	230,233 (4)	<0.001	0.365
	Several times a month	127 (44.1%)	159 (51.5%)	204 (76.4%)			
	Rarely or never	161 (55.9%)	150 (48.5%)	18 (6.7%)			
Fresh fruit	Every day	121 (42.0%)	142 (46.0%)	179 (67.0%)	53,218 (6)	<0.001	0.175
	Several times a week	135 (46.9%)	124 (40.1%)	71 (26.6%)			
	Several times a month	27 (9.4%)	43 (13.9%)	17 (6.4%)			
	Rarely or never	5 (1.7%)	0 (0.0%)	0 (0.0%)			
Berries (fresh, frozen)	Several times a week	0 (0.0%)	17 (5.5%)	28 (10.5%)	93,157 (4)	<0.001	0.232
	Several times a month	238 (82.6%)	155 (50.2%)	173 (64.8%)			
	Rarely or never	50 (17.4%)	137 (44.3%)	66 (24.7%)			
Vegetable and fruit-berry juices	Every day	73 (25.3%)	50 (16.2%)	0 (0.0%)	127,227 (6)	<0.001	0.271
	Several times a week	118 (41.0%)	120 (38.8%)	83 (31.1%)			
	Several times a month	97 (33.7%)	115 (37.2%)	142 (53.2%)			
	Rarely or never	0 (0.0%)	24 (7.8%)	42 (15.7%)			
Tea	Every day	96 (33.3%)	199 (64.4%)	186 (69.7%)	113,527 (6)	<0.001	0.256
	Several times a week	174 (60.4%)	93 (30.1%)	71 (26.6%)			
	Several times a month	1 (0.3%)	5 (1.6%)	10 (3.7%)			
	Rarely or never	17 (5.9%)	12 (3.9%)	0 (0.0%)			
Coffee	Every day	0 (0.0%)	63 (20.4%)	155 (58.1%)	767,470 (6)	<0.001	0.666
	Several times a week	0 (0.0%)	110 (35.6%)	82 (30.7%)			
	Several times a month	0 (0.0%)	79 (25.6%)	30 (11.2%)			
	Rarely or never	288 (100.0%)	57 (18.4%)	0 (0.0%)			
Sweetened carbonated beverages	Every day	35 (12.2%)	44 (14.2%)	78 (29.2%)	59,721 (6)	<0.001	0.186
	Several times a week	168 (58.3%)	166 (53.7%)	118 (44.2%)			
	Several times a month	77 (26.7%)	73 (23.6%)	71 (26.6%)			
	Rarely or never	8 (2.8%)	26 (8.4%)	0 (0.0%)			
Fast food	Every day	0 (0.0%)	101 (32.7%)	130 (48.7%)	402,793 (6)	<0.001	0.483
	Several times a week	30 (10.4%)	105 (34.0%)	104 (39.0%)			
	Several times a month	143 (49.7%)	79 (25.6%)	33 (12.4%)			
	Rarely or never	115 (39.9%)	24 (7.8%)	0 (0.0%)			
